# Potential self-regulatory mechanisms of yoga for psychological health

**DOI:** 10.3389/fnhum.2014.00770

**Published:** 2014-09-30

**Authors:** Tim Gard, Jessica J. Noggle, Crystal L. Park, David R. Vago, Angela Wilson

**Affiliations:** ^1^Department of Psychiatry, Massachusetts General HospitalBoston, MA, USA; ^2^Bender Institute of Neuroimaging, Justus Liebig Universität GiessenGiessen, Germany; ^3^Faculty of Psychology and Neuroscience, Maastricht UniversityMaastricht, Netherlands; ^4^Division of Sleep and Circadian Disorders, Brigham and Women’s Hospital, Harvard Medical SchoolBoston, MA, USA; ^5^Department of Psychology, University of ConnecticutStorrs, CT, USA; ^6^Department of Psychiatry, Brigham and Women’s Hospital, Harvard Medical SchoolBoston, MA, USA; ^7^Institute for Extraordinary Living, Kripalu Center for Yoga and HealthStockbridge, MA, USA

**Keywords:** yoga, self-regulation, stress, executive control, viscerosomatic, top-down, bottom-up

## Abstract

Research suggesting the beneficial effects of yoga on myriad aspects of psychological health has proliferated in recent years, yet there is currently no overarching framework by which to understand yoga’s potential beneficial effects. Here we provide a theoretical framework and systems-based network model of yoga that focuses on integration of top-down and bottom-up forms of self-regulation. We begin by contextualizing yoga in historical and contemporary settings, and then detail how specific components of yoga practice may affect cognitive, emotional, behavioral, and autonomic output under stress through an emphasis on interoception and bottom-up input, resulting in physical and psychological health. The model describes yoga practice as a comprehensive skillset of synergistic process tools that facilitate bidirectional feedback and integration between high- and low-level brain networks, and afferent and re-afferent input from interoceptive processes (somatosensory, viscerosensory, chemosensory). From a predictive coding perspective we propose a shift to perceptual inference for stress modulation and optimal self-regulation. We describe how the processes that sub-serve self-regulation become more automatized and efficient over time and practice, requiring less effort to initiate when necessary and terminate more rapidly when no longer needed. To support our proposed model, we present the available evidence for yoga affecting self-regulatory pathways, integrating existing constructs from behavior theory and cognitive neuroscience with emerging yoga and meditation research. This paper is intended to guide future basic and clinical research, specifically targeting areas of development in the treatment of stress-mediated psychological disorders.

## INTRODUCTION

Research suggesting the beneficial effects of yoga interventions on myriad aspects of psychological health has proliferated in recent years: the extant literature suggests that yoga can improve symptoms of depression, anxiety, stress, post-traumatic stress disorder, and other psychological problems (for reviews, see Kuntsevich et al., 2010; Field, 2011; Balasubramaniam et al., 2012; Li and Goldsmith, 2012) as well as promote well-being, including life satisfaction and happiness (Woodyard, 2011). Many different explanations or pathways for these salutary effects have been proposed, but as yet there is no overarching framework in which to understand them. One useful framework for doing so is that of self-regulation.

Theories of self-regulation are assuming an increasingly central role within various sub-disciplines of cognitive science, psychology, and medicine (see Eisenberg, 2000; Watts, 2000; Gross and Thompson, 2007; McCullough and Willoughby, 2009; Hagger, 2010; Hofmann et al., 2012). Generally speaking, *self-regulation* refers to efforts of monitoring, willpower, and motivation to manage or alter one’s incipient responses and impulses so as to pursue or maintain explicit goals or standards (Luszczynska et al., 2004; Baumeister et al., 2007; Zell and Baumeister, 2013). A focus of contemporary psychotherapy is the development of self-regulation tools to reduce psychological distress and improve well-being. For example, many current cognitive-behavioral treatments focus on using top-down cognitive means of self-regulation, such as cognitive reappraisal, reframing, and goal-setting (e.g., Berking et al., 2008). More recent “third-wave” behavioral and cognitive therapies, such as acceptance and commitment therapy (ACT; Hayes and Wilson, 1994), dialectical behavior therapy (DBT; Hayes et al., 1999), and mindfulness-based cognitive therapy (MBCT; Segal et al., 2002) also target self-regulation, through the development of mindfulness-related skills (Baer, 2005). There have been suggestions in the recent literature that such mindfulness-based approaches may function through both top-down and bottom-up mechanisms of self-regulation (Chambers et al., 2009; Taylor et al., 2010; van den Hurk et al., 2010; Hölzel et al., 2011b; Vago and Silbersweig, 2012; Chiesa et al., 2013; Westbrook et al., 2013). Top-down strategies are thought to occur in more novice meditators, where there is an emphasis on attentional control and thus, top-down executive mechanisms. As the meditation practice deepens, emphasis on interoception increases, evaluation processes decrease across contexts, and bottom-up strategies may be more strongly present. Bottom-up regulation strategies have been described as modulation of emotion-generative brain regions (i.e., limbic) without recruitment of “higher” brain regions (i.e., frontal) that are responsible for cognitive forms of regulation (e.g., reappraisal, suppression; Taylor et al., 2010; van den Hurk et al., 2010; Gard et al., 2012b; Vago and Silbersweig, 2012; Chiesa et al., 2013). More specifically, bottom-up processes involve the influence of peripheral sensory, visceral, cardiovascular, immune, and autonomic input upon central neural processing and mental activities via ascending pathways (Taylor et al., 2010; McRae et al., 2012). Yoga, as we describe the practice here, is a complex, adaptive and widely applicable method of physical and mental training with multiple tools for self-development, and, as we propose, for improving self-regulation through both top-down and bottom-up mechanisms.

In our integrative systems network model, we propose that specific aspects of yoga practice affect self-regulation through tonic feed-forward and feed-back loops across multiple systems, which, in turn, promote psychological and physical health and well-being. *Specifically, we describe how yoga may function through top-down and bottom-up mechanisms for the regulation of cognition, emotions, behaviors, and peripheral physiology, as well as for improving efficiency and integration of the processes that subserve self-regulation.* We begin by contextualizing the system of yoga in historical and contemporary settings. Then we detail how specific components of yoga practice may affect cognitive, emotional, and behavioral systems under stress, potentially resulting in improvements in physical and psychological functioning during practice and in the midst of living everyday life. The hypothesized mechanisms are then integrated in a theoretical model for self-regulation through yoga and finally scientific evidence in support of this model is provided. The intention for this paper is to provide a theoretical framework that can guide future basic and clinical research and specifically guide development in the treatment and prevention of stress-mediated psychological disorders.

## YOGA PHILOSOPHY: A FOUNDATION FOR SELF-REGULATION^1^

In this section a brief historical and philosophical background of yoga will be provided. Yoga, originating from India, is an ancient contemplative practice dating back over 3,500 years, which aims at one thing – to alleviate suffering and promote optimal physical and mental thriving (Cope, 1999; Feuerstein, 2011). In Western contemporary settings, yoga tends to be synonymous with yoga postures, breathing, and some meditation practices. Historically, however, the practice of yoga was understood to be much broader and more comprehensive, including a wider range of techniques to promote wellbeing and balance among mind–brain–body functions. These included paths oriented to service, devotion, intellectual discernment, and meditation, and each offered practices to mitigate suffering and produce higher levels of consciousness (Feuerstein, 2011).

There are many branches of yoga that have developed historically; however, we focus specifically on *Raja* and *Hatha* yoga because of their prevalence in modern practice and their emphasis on developing self-regulation. *Raja* or classical yoga is a system of meditation, while *Hatha* or post-classical yoga followed *Raja* yoga and elaborated upon postures and breathing techniques largely to prepare for meditation. Accordingly, modern yoga practitioners—who practice for purposes beyond physical fitness—study *Hatha* yoga within the context of *Raja* yoga (Vivekananda, 2001). We also focus on *Raja* yoga because many of its components can be linked to modern physical and mental self-regulation concepts. As will be discussed in more detail in the next section, these components can be linked to self-regulatory processes such as goal-setting (top-down ethics), observation of one’s behaviors in relation to these goals (top-down attentional processes and bottom-up sensing during postures, pranayamas, and meditation) and cultivation of the ability to override incipient responses in order to move closer to goals (including ethical motivations; McCullough and Willoughby, 2009; Zell and Baumeister, 2013).

The focus of *Raja* yoga, as outlined by Patanjali (author of the *Yoga Sutras,* a historic text of *Raja* yoga, circa start of the common era), was primarily cognitive. Patanjali described yoga as the stilling of distorted fluctuations or ruminations in the mind, which are the sources of suffering (Vivekananda, 2001; Cope, 2006). The multicomponent process of *Raja* yoga is aimed toward training the mind to be effortlessly quiet, focused, and self-aware. These cognitive goals of *Raja* yoga overlap with some goals of other meditative traditions such as Buddhism (Feuerstein, 2011), from which the modern concept of mindfulness has sprung (Kabat-Zinn, 1994; Bodhi, 2011). Some scholars in the humanities consider Patanjali and Shakyamuni Buddha as contemporaries. According to them, in the process of his self-transformation, the Buddha studied and mastered Upanishadic yoga techniques, and his teachings were influenced by these experiences (Feuerstein, 2008; Gombrich, 2009; Gethin, 2011), but see (Bronkhorst, 2007, 2013). Furthermore, the cross-pollination of yoga and Buddhism is most evident in the overlap of Vajrayana Buddhism and *Hatha* yoga (Feuerstein, 2011). A modern example of this overlap is the mindfulness-based stress reduction (MBSR) program, which includes some Hatha yoga postures (Kabat-Zinn, 1990). Although much of the mindfulness-based practices emphasize the mental form of training, some elements of yoga asana and pranayama remain in MBSR. Interestingly, one study of participants from nine different MBSR courses found yoga practice time to be more strongly correlated with self-reported improvements in mindfulness, perceived stress, anxiety, and psychological well-being than formal sitting meditation time during the 8 weeks (Carmody and Baer, 2008).

Patanjali’s *Raja* yoga offers eight different groups of practices aimed toward self-regulation. In Patanjali’s *Yoga Sutras*, these different groups of practices are called the eight limbs (**Table 1**), and include: moral observances (ethics when interacting with others); self-discipline (ethics geared toward the self); physical postures and exercises; breath regulation; sensory withdrawal (minimizing sensory input); concentration (effortful, focused attention); meditation (effortless, unbroken flow of attention), and self-transcendence (Stone, 2009). Collectively, the eight limbs may be conceptualized as methods to regulate emotions, thoughts, or behaviors and to increase well-being (Cope, 2006). The diversity of limbs allows students to begin yoga by working with practices that are most appealing and accessible, often the physical postures for Western students.

**Table 1:** Components of classical yoga (the eight limbs of Patanjali’s *Raja* yoga).

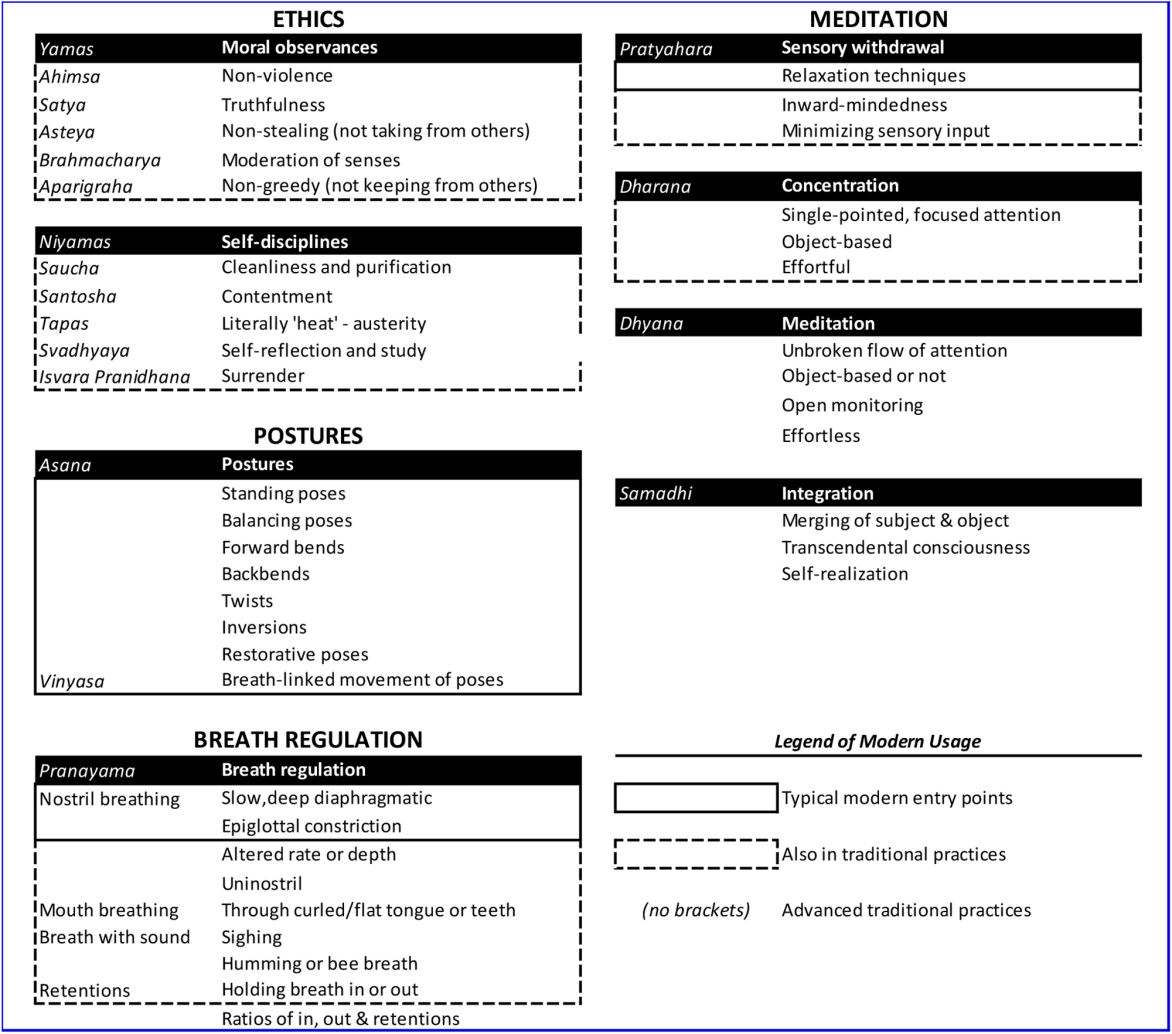

## YOGA’S TOOLS FOR SELF-REGULATION: HISTORICAL AND MODERN INTERPRETATIONS

In this section, we describe the components of *Raja* yoga on which we base our proposed model of self-regulation. To this end we linked classical components of yoga to modern, scientific concepts. We have grouped the eight limbs under the following four categories referred to herein as “process tools” (**Table 1**), because a combination of these four categories encompasses most modern yoga classes (Field, 2011) and most research on yoga emphasizes these particular practices (Li and Goldsmith, 2012): (1) Ethics, based on the two ethical limbs (moral observances (Sanskrit*: yama*) and self-disciplines (Sanskrit*: niyama*)); (2) Postures (Sanskrit: *asana*); (3) Breath regulation (Sanskrit*: pranayama*); and (4) Meditation, including the four meditative limbs (sensory withdrawal (*Sanskrit: pratyahara*), concentration (Sanskrit*: dharana*), meditation (Sanskrit*: dhyana*), and a deep level of concentration or absorption also described as self-transcendence (Sanskrit*: samadhi*)).

### ETHICS (YAMA AND NIYAMA)

On the foundation of the yogic path of self-regulation lie ethical and moral precepts, which are specific examples of the standards or guidelines that contribute to self-control suggested by Zell and Baumeister (2013). These ethical precepts are contained in the first and second limb of Patanjali’s eightfold *Raja* yoga path, namely *yama* and *niyama,* respectively. *Yama* refers to ethics regarding the outside world, and therefore is particularly important in social contexts. It comprises non-violence (Sanskrit: *ahimsa*), truthfulness non-stealing, moderation of senses, and greedlessness. *Niyama* refers to ethics regarding the inner world. It comprises purification or cleanliness and contentment, austerity (Sanskrit: *tapas*), self-reflection and surrender or devotion to something greater than oneself. As such, the ethics suggested in yoga are devoid of religious connection—they are not based on moral value judgments of right and wrong—but are rather seen as actions that help to quiet an overactive mind, regulate emotions, and enhance prosocial and skillful behaviors (Cope, 2006).

### POSTURES (ASANA)

In Patanjali’s *Yoga Sutras*, the limb of *asana* is defined as steady and comfortable posture (Cope, 2006). Physically challenging postures are further described to be sustained through the fluctuations of the mind (Faulds, 2005). Postures are one of the most commonly utilized yoga practices in modern interpretations. Historically, postures were used to physically control the body in preparation for controlling the mind in meditation for extended periods of time (Feuerstein, 2011). A common premise behind modern yoga classes is that practicing various postures may help to reduce physical and emotional stress. A typical yoga class will include a series of postures targeting different parts of the body. For example a class might include forward and backward bends, twists, standing poses, and balancing poses (**Table 1**). Modern and historic yoga practice manuals such as *Light on Yoga* (Iyengar, 1995) often suggest a connection between emotional states, physical health, and postures. Although this link has not scientifically been established yet for any particular poses (or set of poses) specifically, there is evidence linking posture, emotion, and mental health (Michalak et al., 2009, 2011, 2014). We attempt to address these hypothesized benefits in the following section.

### BREATH REGULATION (PRANAYAMA)

The Sanskrit word *pranayama* is composed of the word *prana*, which translates to breath as a life-sustaining force, and the word *ayama* which translates to freedom or release. *Pranayamas* are a series of specific techniques to control the breath in order to allow the breath and life force to flow freely (Sovik, 1999). Traditionally, two benefits of *pranayama* are described to help the practitioner down-regulate arousal and increase awareness of the interaction between the body and the mind (Sovik, 1999). Similar to *asana* as preparation of the body for meditation, *pranayama* is meant to prepare the mind for meditation. *Pranayamas* differ from normal breathing on a number of dimensions, including the duration of the in breath, the out breath, the holding of the breath, and the ratio of these. All *pranayamas* involve diaphragmatic breathing, mostly deep and slow in quality through the nose (Jerath et al., 2006). Popular pranayama techniques include deep, even, three-part inhales and exhales, alternate nostril breathing, forceful expulsion of breath using the diaphragm and abdominal muscles, and slow diaphragmatic breathing with partial closure of the glottis creating an audible sound of rushing air described “like an ocean.” For a more extensive overview of *pranayamas* see Singh et al. (2009).

### MEDITATION (PRATYAHARA, DHARANA, DHYANA, SAMADHI)

Postures and breathing practices are traditionally described to support and foster meditation practices (Faulds, 2005). In the yoga tradition, the concentrative meditation techniques described in *Raja* yoga help the practitioner begin to see the conditions that lead to mental and emotional suffering (fluctuations) and the conditions that remedy suffering (i.e., mental stillness). Suffering is further described as a time when the mind is in an aﬄicted state—either grasping onto an experience, not wanting to let it go, or experiencing aversion, trying to push some object of experience away with force. In both cases, how one relates to one’s inner experience will create either more or less suffering. Such suffering can also be described as a mental state that prevents the mind from seeing reality without emotional bias. The ability to see reality clearly without bias, through meditation practices, is a revered tool of self-regulation within yoga (Cope, 2006) and similarly in mindfulness traditions (Vago and Silbersweig, 2012).

The various forms of meditation in the *Raja* yoga tradition are considered key tools in the regulation of the mind (**Table 1**). Specifically, *Raja* yoga includes a set of concentration practices. For example, sensory withdrawal (*Sanskrit*: *pratyahara*) involves techniques to minimize external distractions from sensory information, facilitating a calm mind and allowing attention to turn inward. Many modern yoga classes conclude with supine rest pose (*Sanskrit: savasana*), the body relaxed and eyes closed. *Pratyahara* techniques include guided relaxation (e.g., *yoga nidra*), which serve as an invitation for students to draw their attention to their inner experience.

The next phase of meditation is called *dharana*, coming from the Sanskrit word “dhr” or to hold tight. In *dharana* the practitioner aims to focus the mind on a single object of meditation such as the breath, a point on the body or an external object (e.g., candle flame) and attempts to maintain focus on that object. At this stage of practice, focused attention requires effort as the mind repeatedly wanders. A key goal of *dharana* is to minimize mind wandering (similar to focused attention meditation (see Benson, 2000; Lutz et al., 2008; Hasenkamp et al., 2012), such that when one realizes the mind has turned away from the object of meditation, the mind is continually brought back to the object.

Occasionally the mind ceases wandering, and meditation shifts into what *Raja* yogis consider more advanced meditative practices, *dhyana*. With time and practice, wandering decreases, as does effort to maintain focus, and an unbroken chain of awareness rests on the object of meditation (Telles et al., 2010; Feuerstein, 2011). As this mastery occurs, there is less effort to keep the mind on the object of concentration and a natural concentrative ease begins to happen. The mind begins to become completely absorbed in the object of attention and a sense of union with the object of attention can begin to occur (Cope, 2006). An analogous process is the psychological flow state experienced by advanced musicians and athletes (Csikszentmihalyi, 1997; Khalsa et al., 2009).

This leads to the final meditative limb, *Samadhi,* which represents transcendent states of conscious awareness and absorption associated with a non-dual subject/object distinction (Josipovic, 2010; Travis and Shear, 2010). *Samadhi* has been described as an experience of no conceptualization, where the object is known directly, beyond name and form. This state of meditation offers a deep sense of interconnection and “sameness” with all phenomena (Cope, 2006). While these experiences of *dhyana* and *samadhi* are said to have profound effects on the mind, these advanced practices are not commonly taught in modern styles of yoga.

Rather than being taught as explicit practices, these meditative limbs are often offered as a process of meditative focus throughout practice. For example, Kripalu yoga, a modern style of *Raja* and *Hatha* yoga, emphasizes witness consciousness, or the observation of experience without reaction (Cope, 2006). Witness consciousness can be likened to the state of mindfulness, often described as “paying attention in a particular way: on purpose, in the present moment, and nonjudgmentally” (Kabat-Zinn, 1994, p. 4). In yoga class, students are guided into a pose, invited to deepen their breath, and then to witness (be mindful of) their experience. The specific limbs that fall under the category of “meditation” are all essential factors that are described to co-arise through the prescribed practices and with the other limbs for progression and mastery. Importantly, witness consciousness can be thought of as a critical mental factor that arises with the other factors serving yoga, but also with the quality of monitoring the development and balance of the other limbs.

The modern use of yoga tends to synergize some of the meditative techniques with postures, breathing, and ethics (**Table 1**). As such, yoga is a unique practice because it offers a variety of complementary tools with which the practitioner can increase self-regulation.

## A THEORETICAL MODEL OF SELF-REGULATION THROUGH YOGA PRACTICE: HOW CURRENT COGNITIVE NEUROSCIENCE AND PSYCHOLOGICAL THEORY CONTRIBUTES TO REVEALING MECHANISMS OF YOGA

**FIGURE 1:**
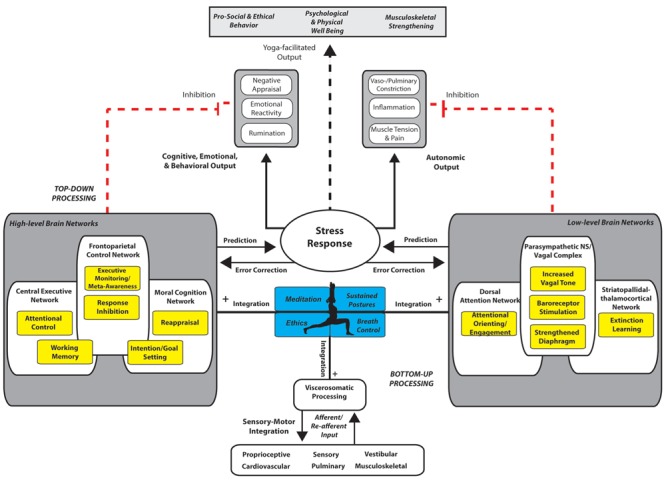
**Systems network model of yoga for optimizing self-regulation.** The major limbs of yoga are represented in blue boxes as a skillset of four process tools: ethics, meditation, breath regulation, and postures. Application of these skills (limbs of yoga) across cognitive, emotional, behavioral, and autonomic domains in the context of physical and emotional stress is proposed to generalize to similar challenges off the yoga mat and in everyday life. Together, these tools of yoga improve the efficiency, bidirectional feedback, and integration (+ black lines) between high- and low-level brain networks, and afferent and re-afferent input from interoceptive processes (e.g., multi-sensory, proprioceptive, vestibular, cardiovascular, pulmonary, musculoskeletal) in the context of stress. Through an emphasis on interoception and bottom-up input, integration facilitates inhibition (red lines) of maladaptive forms of cognitive, emotional, and behavioral output as well as autonomic output associated with stress. Efficiency improves the communication and flexibility between brain and bodily systems to inform behavioral output. Yoga’s four tools are described to involve particular regulatory processes associated with each set of brain networks (indicated in yellow boxes). With mastery of practice, regulatory processes become more automatized, requiring less effort to initiate when necessary and terminate more rapidly when no longer needed. A central executive network supports top-down mechanisms of attentional control and working memory allowing monitoring for proper goal-directed behavior followed by self-correction if needed. A FPCN supports executive monitoring, meta-awareness, reappraisal, and response inhibition mechanisms. A moral cognition network supports motivation and intention setting associated with self-care and prosocial behavior. The dorsal attention network helps to support attentional orienting, and engagement. Hypothlamic–pituitary–adrenal (HPA) axis communication with brainstem vagal efferents support parasympathetic control and homeostasis across systems. A striatopallidal–thalamocortical network is responsible for facilitating extinction learning and reconsolidation of maladaptive habits into behavior that is aligned with intentions and outcomes into adaptive habits. Dotted lines represent new, adaptive pathways for responding to stress. A focus toward bottom-up processes facilitates a shift toward perceptual inference rather than active inference, and improves prediction and error correction processes, thus supporting optimal self-regulation.

As discussed above, yoga can be broken down into a skillset of four tools for self-regulation: (1) ethical precepts, (2) sustained postures, (3) breath regulation, and (4) meditation techniques. Here we propose a model (**Figure 1**) that describes how this skillset may facilitate self-regulation and results in psychological and physical well-being. In this model, we propose how yoga skills facilitate bidirectional feedback and improve integration and efficiency of high-level (e.g., central executive network, frontal–parietal control network) and low-level brain networks (e.g., autonomic systems, vagal complex, striatopallidal–thalamocortical network) along with viscerosomatic, musculoskeletal, cardiac, respiratory, and sensory information coming from the periphery (see **Table 2** for description of networks). As depicted in **Figure 1**, maladaptive cognitive, emotional, and behavioral output (e.g., negative appraisal, emotional reactivity, rumination), as well as physiological output initiated by lower-level brain systems (e.g., sympathetic-related vaso- and pulmonary constriction, inflammation, muscle pain/tension) that disrupt homeostatic conditions across bodily systems (including cardiovascular, neuroendocrine, and musculoskeletal) are extinguished and replaced with more adaptive output to the challenging demands of stress in the context of practice and in more generalizable settings. Integration between top-down and bottom-up processes is also proposed to improve accuracy of prediction and error correction mechanisms associated with the stress response across domains, resulting in further improving accuracy in detecting and efficiently responding to perceived threats and reducing consequences of prolonged stress exposure. Finally, the model poses that regular practice is partially motivated and enhanced by a particular set of ethical beliefs promoting benefits toward oneself and others, including direct reward resulting from the practice. Thus, increased activation of a higher level moral cognitive network (see **Table 2**) is hypothesized to be associated with improved ethical skills. It is understood that placebo-related mechanisms may also operate to fuel effective top-down control and motivation; however, such mechanisms remain unclear. Nonetheless, motivation provides realistic goals and positive intentions to fuel approach behavior and provides the scaffolding to support the ethical framework built in to the practice.

**Table 2:** High-level and low-level brain networks.

**Central executive network**
Dorsal parietal cortex	Superior parietal lobe (sPL)	Dorsal frontal cortex
**Frontoparietal control network**
Frontopolar cortex	Dorsolateral prefrontal cortex (dlPFC)	Dorsomedial prefrontal cortex (dmPFC)
Anterior cingulate cortex (ACC)	Anterior inferior parietal lobe (aIPL)	Anterior insular cortex (AIC)
Temporo-parietal junction (TPJ)	Ventrolateral prefrontal cortex (vlPFC)	
**Moral cognition network**
Ventromedial prefrontal cortex (vmPFC)	dmPFC	Posterior cingulate cortex (PCC)
Precuneus	Middle temporal gyrus (MTG)	TPJ
**Dorsal attention network**
Frontal eye fields (FEF)	Ventral premotor cortex (vPMC)	sPL
Dorsal parietal cortex	Supplementary motor area (SMA)	Ventromedial posterior nucleus–thalamus (VMpo)
Pulvinar	Superior colliculus (sc)	
**Striatopallidal–thalamocortical network**
Orbitofrontal cortex (OFC)	ACC	Dorsal striatum
Thalamus	Pallidum	VTA/SN

### TOP-DOWN INFLUENCES ON SELF-REGULATION

Here, we propose top-down self-regulatory mechanisms of yoga include control of intentional/motivational drive (e.g., goal-setting and maintenance), working memory, attention, executive monitoring, response inhibition, reappraisal, and meta-awareness (see yellow boxes within high-level brain networks in **Figure 1**). Such control signals include generation and maintenance of attention on the object of practice, which includes a continuous focus on an object of visual attention (e.g., point in space), aspect of the breath, or interoceptive feedback from body sensation or mental activity. Furthermore, cognitive reappraisal of feedback from the body is proposed to facilitate inhibitory tone toward maladaptive cognitions, emotions, or behavior.

Yoga is often called “meditation in motion” (e.g., Khalsa et al., 2009) for its highly focused attention during bodily movements. Attention is often directed to a specific part of one’s body (e.g., ball of foot, fingertips) or an external point (Sanskrit: *drishti*); similarly, in pranayama, practice involves focusing on the breath as it moves through the body. The focus of one’s gaze on one point facilitates the “withdrawing” of the sense of vision away from distraction, while the focus of one’s attention inward and on body sensation contributes to the “withdrawing” of the other senses from distractions. Distraction is the loss of selective attention and focus on sensory experience outside of a single point or prescribed focal points. Rapid disengagement from distraction and re-engagement on the object of focus is also believed to be a top-down strategy (Koster et al., 2011), although early attentional filtering may involve more bottom-up sensory processing (Kerr et al., 2013). Attentional stability in the context of stressors induced through yoga practice is thought to reduce negative forms of appraisal and ruminative thought processes by maintaining awareness and attentional engagement with sensation in the body (McCall, 2007). This form of intentional concentration on particular sensations and not others is critical to cognitive flexibility of attention and inhibitory control – the ability to sustain attention on meaningful information and disregard irrelevant information from the external and internal environment. Sensory withdrawal, one of the originally described “limbs” of Patanjali’s yoga, is therefore conceptualized here as contributing to cognitive factors of selective attention and response inhibition, further reducing autonomic reactivity and habitual tendencies to respond to external and internal experience in maladaptive ways.

A central executive network has been found to be most consistently active for the described forms of explicit attention and cognitive regulation, including the dorsal parietal cortex along the intraparietal sulcus, extending dorsomedially into the superior parietal lobe, and anteriorly toward the post-central sulcus, and the dorsal frontal cortex (Corbetta and Shulman, 2002). In addition to the central executive network, a fronto-parietal control network (FPCN) is also proposed to help facilitate executive monitoring (of internal and external information) and meta-awareness, guidance of decision making, salience detection, and integration of information from the external and internal environment (Seeley et al., 2007; Vincent et al., 2008). This network includes the frontopolar cortex (FPC), dorsolateral and ventrolateral prefrontal (dlPFC, vlPFC), dorsomedial superior frontal/anterior cingulate cortex (ACC), anterior inferior parietal lobe (aIPL), temporoparietal junction (TPJ), and anterior insular cortex (AIC; Vincent et al., 2008; Spreng et al., 2013). One central aspect of yoga practice thought to facilitate the engagement of this network is meditation and its associated skills for fostering stable attention through active inhibition of specific brain regions responsible for processing internally generated contents of our attention (e.g., default mode network DMN) and facilitation of executive monitoring and present-centered sensory processing.

Meta-awareness or self-awareness (Zell and Baumeister, 2013) may be another mechanism through which yoga improves self-regulation. Increased meta-awareness would allow individuals to clearly compare their current status *vis à vis* their goals and to make any necessary behavioral adjustments. Meta-awareness is often cited along with psychological distancing (Ayduk and Kross, 2010) or decentering (Fresco et al., 2007), a process that allows the individual to uncouple the sensory experience from the “narrative” self and gain perspective of one’s world-view and habits, and increases the likelihood of behavioral change (Critchley et al., 2004; Vago, 2014). Furthermore, the de-centered perspective allows one to experience thoughts and emotions in terms of their subjectivity (rather than an assumed validity) and their transient nature (rather than their assumed permanence; Fresco et al., 2007). Such awareness is thought to override short-term impulses in favor of long-term considerations, effectively improving one’s ability to reflect upon the past and use that information accurately to anticipate the future, and to more accurately assess the present (Baumeister et al., 2011). This form of awareness has also been shown to facilitate prediction of error detection and correction, and as a result, more rapidly improve behavioral correction processes when regulating emotional responses to stress (Compton et al., 2008).

With support from the neuroscientific literature (Critchley et al., 2004; Craig, 2009; Taylor et al., 2010), our model suggests that meta-awareness of interoceptive experience facilitates integration across primary sensory, visceral, homeostatic, environmental, hedonic, and social levels via feedforward and feedback between higher-level and lower-level brain networks. For example, there is good evidence that salience-specific nodes of the FPCN (ACC, and insula), interact to develop modulatory influence over viscerosomatic afferent activity arising through the spinal–thalamo-cortical pathway (Craig, 2004). The insula specifically processes interoceptive input in a hierarchical fashion – in a posterior to anterior direction (Craig, 2009), with more basic sensory and homeostatic information in the posterior and more salient, hedonic, and social information in the anterior. Such interaction and integration are proposed to result from the strengthening of the executive monitoring system similarly to mindfulness-based meditative practices (Vago and Silbersweig, 2012; Desbordes et al., 2014). Mindfulness practice encourages practitioners to take a meta-cognitive view of their experience, to notice the experience without judging it or modifying it and is thus, a form of self-regulation. Yoga offers a way to practice mindful awareness in this way, described above as “witness consciousness.”

Self-regulation of emotion refers to the management of the felt experience of emotions, both positive and negative in valence, its behavioral expression, and associated autonomic output, through cognitive reappraisal and through a process of non-appraisal that involves awareness alone, a form of attentional control that does not involve evaluation or judgment (Davidson and Irwin, 1999; Critchley et al., 2003; Vago and Silbersweig, 2012). We theorize that emotional stability and rapid recovery from perturbation arises from either strategy. Similarly to the difference between beginning and experienced mindfulness meditators, non-appraisal is believed to be more advanced and more likely in experienced yoga practitioners than in novices (Taylor et al., 2011). The rapid recovery from emotional perturbation has been referred to as equanimity in the Buddhist and secular mindfulness literature. Equanimity, defined as an even-minded mental state or dispositional tendency toward all experience, regardless of affective valence or source (Desbordes et al., 2014), is proposed to be a skill that is manifested through efficient feed-forward and feedback between somatic, autonomic, viscerosensory, and high-level brain network activity that continually manages stressors that arise on and off the yoga mat.

Positive reappraisal is yet another important cognitive and emotion regulation skill that can be enhanced by yoga. Positive reappraisal has been proposed to mediate the stress-reductive effect of mindfulness through an upward spiral process (Garland et al., 2011). For example, much of yoga practice involves learning to reframe experiences (e.g., from discomfort to sensation), teaching a more objective, observational, and non-judgmental stance to one’s experience (Garland et al., 2011). Other reappraisal resources can be acquired through yoga practice. For example, in the course of yoga classes, teachers often instruct students about deeper aspects of yoga philosophy, such as compassion and impermanence, which are useful in appraising or reappraising situations as less stressful (Nyklíček, 2011). Kripalu yoga promotes a technique of “riding the wave” (Faulds, 2005), which involves reappraisal and behavioral change that allows toleration of discomfort – reframing the discomfort of muscle fatigue to a temporary sensation that will pass. Recent theories on self-regulation postulate that explicit reappraisal strategies can reduce the negative impact of prolonged and repeated high stress-related neuroendocrine activity (Koole et al., 2011; Geisler et al., 2013). Reductions in perceived stress lead to conserved cognitive resources and less prolonged (or perseverative) negative emotion (e.g., anger, frustration) along with peripheral effects (e.g., increased vasodilation) that improve outcomes (Jamieson et al., 2012; Keller et al., 2012). The development of equanimity and meta-awareness may also improve self-other interactions and therefore contribute to ethically informed pro-social behavior.

Yoga is also proposed to enhance behavioral forms of self-regulation through goal-directed influence over one’s behavior “on and off the yoga mat.” This is a topic particularly relevant to health psychology (Mann et al., 2013), such that health-promoting behaviors are more likely once a practitioner has committed to the system and practice of yoga and has an attitude supporting “readiness” or “willingness to change.” For example, readiness to change has been found to be a significant factor driving clinical change in addiction settings (DiClemente et al., 2004). Regulation is proposed to occur via continual adjustment and guidance of one’s behavior (e.g., maintain a particular body position even if one’s muscles ache and the impulse is to relax) in pursuit of ethically motivated actions associated with self-care, health-promoting behavior, and pro-social interactions (e.g., increased empathic behavior). Behavioral strategies may also limit exposure to stressful situations. For example, the individual may avoid potentially stressful situations and employ proactive coping strategies to prevent them (Aspinwall and Taylor, 1997) or engage in socially adaptive behaviors (e.g., seek social support; Geisler et al., 2013), both of which are behavioral forms of self-regulation. In addition, yoga practitioners may move toward more healthy lifestyles (e.g., involving less substance abuse, decreasing their risk for substance-related accidents, healthy eating habits; Khalsa et al., 2008; Carei et al., 2010). Thus, behavioral regulation also encompasses a range of inhibitory response behaviors, such as overriding impulses, habits, or cravings, employing approach-focused coping (van Gaal et al., 2011), and facilitating ethical behavior. The effects of yoga on cravings and aversions remain unclear and pose an interesting question for future research.

Yoga can be considered a “wisdom-based” contemplative practice (Cope, 2006), providing an ethical framework that is based on discernment of right and wrong action. Particular ethical precepts of yoga practice are rarely taught formally in Western yoga classes, but they may be conveyed in more indirect ways, such as through modeling, suggestions for goal-setting, or technique instructions that may include attitude and awareness of self and other. In Kripalu yoga, for example, gentleness and respecting one’s own boundaries are important aspects of the instructions. These instructions may convey the ethics of non-violence (*ahimsa*) and contentment (*santosha*). Conceptually these two ethics may be related to (self)-compassion, which has been shown to be cultivated through yoga and which mediates the positive effects of yoga on well-being and perceived stress (Gard et al., 2012a). Shapiro et al. (2012) argue that while ethics form the foundation for the cultivation of other mental qualities as described in yoga as well as in Buddhist traditions, the cultivation of states of discernment and meta-awareness even in the absence of explicit training in ethics would affect moral reasoning and decision making in an implicit way. Such changes have been suggested to be mediated by mechanisms of sensory–perceptual clarity, enhanced compassion, values clarifications, and increased emotional awareness and regulation (Eisenberg, 2000). Indeed, improved moral reasoning and decision making along with improvements in witness consciousness, which includes states of discernment like mindfulness, have been reported two months after a MBSR intervention, suggesting that contemplative interventions that incorporate mindfulness and yoga can result in development of these ethical aspects without explicit teaching of them (Shapiro et al., 2012). Similarly, Thiel et al. (2012) propose that emotion regulation, self-reflection, and information integration are three of the four strategies affecting ethical decision making. Interestingly, all of these three constructs are thought to be affected by MBSR and trait mindfulness (Hölzel et al., 2011b), a trait found to be cultivated through yoga (Gard et al., 2012a).

Numerous neuroimaging studies have investigated the neural underpinnings of moral decision making. A recent meta-analysis revealed that moral cognition involves a brain network including ventromedial prefrontal cortex (vmPFC), dorsomedial PFC, posterior cingulate cortex (PCC), precuneus, middle temporal gyrus, and TPJ (Bzdok et al., 2012). This network has several nodes in common with networks supporting theory of mind and empathy, suggesting that moral cognition and prosocial behavior involve integration of both socio-cognitive and socio-affective networks (Bzdok et al., 2012). Interestingly, MBSR has been shown to improve moral cognition (Shapiro et al., 2012) and empathy (Shapiro et al., 2011) and modulate structures associated with perspective taking (Lazar et al., 2005; Hölzel et al., 2011a,b). Likewise, we propose yoga practice engages this network to influence and regulate behavior and prosocial skills.

### BOTTOM-UP INFLUENCES ON SELF-REGULATION

We emphasize that as opposed to classical psychotherapeutic approaches for self-regulation that rely only on cognitive strategies, mind–body approaches including yoga involve both top-down and bottom-up mechanisms (see Taylor et al., 2010; Chiesa et al., 2013). Successful self-regulation through bottom-up mechanisms is proposed to facilitate improved function in a network of low-level brain structures (associated regulatory processes indicated by yellow boxes in **Figure 1**) responsible for peripheral physiological activity and reorganization, including stimulation of somatic, visceral, and sensory receptors, extinction processes, and integration with homeostatic physiology. Specifically, we propose yoga practice involves coupling between the body and environment that facilitates embodied viscerosomatic integration and action (see bottom of **Figure 1**). Building upon past theories of somatic processing which emphasize the representation of emotional states in subcortical maps as it interacts with the environment (Damasio, 2010), here we similarly emphasize embodied, situated forms of processing. Viscerosomatic information from the body, including primary sensory and homeostatic information, is proposed here to be continually and reciprocally integrated with motor output and ongoing cognition during sustained body postures, diaphragmatic breath control, attentional control, and dynamic patterns of representative neural activity. The shift in attentional orientation toward the body and significant ongoing reciprocal communication between sensory-motor afferent and re-afferent input supports a model for integration and embodied cognition and behavior (Wilson, 2002; Taylor et al., 2010; Schmalzl et al., 2014).

Embodiment implicitly refers to action coupled with and not separate from the bodily experience. Merleau-Ponty (1996) refers to this experience as, “I am not in front of my body, I am in my body, or rather I am my body.” Yoga may offer additional tools of embodiment (e.g., explicitly inducing gross levels of physical sensations) for some populations that sitting meditation alone cannot provide. For example, a few studies have suggested how mindful yoga may be more suited for those with chronic illness or for patients with psychopathology and a history of trauma (van der Kolk et al., 2014). Yoga may support changes in chronic illness schema by challenging beliefs about the limits of one’s physical body. As students practice poses and bring awareness to sensation in the body in ways they initially may have felt they could not, they may come to see that while some aspects of their bodies are limited, other parts of the body are still healthy (Hamilton et al., 2006). van der Kolk (2006) described how movement and touch-based practices that focus on the body [embodiment] have demonstrated some level of effective self-regulation, including Feldenkrais, Rolfing, the F. M. Alexander technique, somatic experiencing, among others. van der Kolk (2006) further suggests it is the trauma patients who specifically have learned helplessness and immobility associated with their emotional trauma. Frewen and Lanius (2006) describe how individuals with PTSD are often unable to up regulate their level of arousal during periods of anhedonia or “emotional numbness,” which further prevents effective regulation in the context of affective arousal or distress. Thus, body movement during yoga practice and activation of motor and inhibitory control circuits like the striatopallidal–thalamocortical loops in the context of physical arousal may be a more effective way of triggering exposure, extinction, and adaptive reconsolidation of emotions. With embodiment, we propose sensory and perceptual faculties are sharpened – a sense of clarity or greater phenomenal intensity in which sensation is experienced emerges.

Early attentional filtering, a form of primary sensory processing of the environment, may also be affected by yoga practice. For example, a dorsal attention network of brain structures [i.e., frontal eye fields (FEF), ventral premotor cortex (vPMC), superior parietal lobe (sPL), intraparietal sulcus (IPS)] selects information from the sensory world (with direction from top-down influences) and is most associated with covert and overt shifts of spatial attention, eye movements, and hand–eye coordination directed from brainstem nuclei and pre-motor areas (Corbetta and Shulman, 2002; Vincent et al., 2008). Low-level brain networks for this process are typically described to function under conscious awareness, contributing to affective biases of perception (Todd et al., 2012), but also to neuro-visceral integration as described by Thayer and Lane (2000). For example, the “experiential enactive self” network (e.g., supplementary motor area (SMA), ventromedial posterior nucleus of the thalamus (VMpo), pulvinar, posterior insula, and superior colliculus (SC; Vago and Silbersweig, 2012) has been described to contribute to perceptual biases, and to be modulated by mindfulness practice. The posterior insula, a primary node in this network, has been implicated in processing the most basic primary interoceptive information along with homeostatic sensory-motor information (Craig, 2009). It remains unclear how these networks specifically interact as a result of yoga training, but we hypothesize that they are reconditioned to facilitate engagement with body sensations, reduce bias and be more functionally integrated with viscerosomatic input, executive control, and adaptive motor output.

In the context of yoga-induced physical stress and associated emotional reactivity, there is a coordinated set of homeostatic responses involving the interaction among the nervous, endocrine, and immune systems (McEwen, 1998). Overactive or inefficiently managed homeostatic responses can lead to cumulative wear and tear on the body and brain (Sterling and Eyer, 1988; McEwen, 2004). Pathophysiological processes with unfavorable psychopathological outcomes may occur given the following prolonged conditions: (1) if the release of neuroendocrine mediators are not effectively terminated (in the presence or absence of a stress-related physiological challenge), (2) if there is failure to habituate to repeated stress-related challenges, or (3) if there is a failure to mount an adequate response to the stressor (McEwen, 2004). Within this framework, we propose that yoga affects self-regulation through parasympathetic control, in part by physiologically reducing prolonged emotional reactivity and associated autonomic responses, both in terms of acute forms (e.g., during/immediately following a yoga practice) and by shifting a physiological set point for baseline reactivity over repeated, long-term yoga practice. In other words, it may take more physical stress or more intense emotional reactivity to induce a typical “stress response” in addition to promoting parasympathetic tone more rapidly in response to stress. The current model predicts physiological mediators such as the catecholamines (e.g., epinephrine, norepinephrine) from the adrenal medulla, glucocorticoids (e.g., cortisol) from the adrenal cortex, pituitary hormones (e.g., ACTH, prolactin, growth hormones), and inflammatory cytokines (e.g., IL-1, IL-6, TNF-α) from cells of the immune system are modulated directly in various bodily tissues and organs to facilitate rapid homeostasis across all bodily systems effectively managing the bodily responses to the stress of challenging postures and distraction. Furthermore, there is some evidence that meditative components of yoga can increase levels of brain-derived neurotrophic factors (BDNF; Balasubramaniam et al., 2012), a growth factor thought to help support the survival of existing neurons and counteract degenerative effects of inflammation by encouraging the growth and differentiation of new neurons (Huang and Reichardt, 2001). Properly managed stress through the challenges of focusing attention with stillness, balance, and breath control across repeated yoga practice has the potential to promote neurogenesis, dendritic arborization, and increased synaptic connectivity, and can be reinforcing by enhancing neural activity in the “pleasure pathways” of the nervous system (i.e., basal forebrain; Chrousos and Gold, 1992b; Logan and Barksdale, 2008). This change in homeostatic responsivity is a form of skillful optimization of autonomic control to keep arousal at lower levels, helping the practitioner stay relaxed with less effort, and facilitate recovery of bodily systems under stress. Such “calming” effects by contemplative practices have been previously described as the “relaxation response” (Benson, 2000; Dusek and Benson, 2009) and refer to what is described in the extant yoga literature as “steadying the mind.” Similarly, it has been suggested that yoga promotes continual neurovisceral feedback and a mechanism of increased inhibition of sympathetic responses post-arousal via branches of the 10th cranial nerve, the vagus nerve, and specific parasympathetic nuclei in the medulla (Streeter et al., 2012). In contrast the to the “relaxation response,” the “stress response” refers to both the sympathetic output and cognitive, emotional, and behavioral output that are associated with detection of threat (Benson, 2000).

Parasympathetic responsivity, a form of autonomic nervous system regulation, is described to manifest in cardiac variability or vagal tone (Porges, 1995; Thayer and Lane, 2000). Cardiac vagal tone, measured by respiratory sinus arrhythmia (RSA) and heart rate variability (HRV), indexes the efficiency of central–peripheral feedback mechanisms (Thayer and Lane, 2000). Specifically, RSA is a cardiorespiratory phenomenon that indexes the influence of respiratory frequency and depth of ventilation (i.e., tidal volume) on vagal control of heart rate (HR); whereas, HRV refers to the peak-to-peak variability between successive beats (Grossman and Taylor, 2007). Yoga practice may facilitate high vagal tone (Streeter et al., 2012), which is associated with greater behavioral flexibility in a changing environment and can manifest in decreases in low-frequency HRV, increases in high-frequency HRV, and increased RSA. Vagal tone refers to the activity of the vagus nerve, and its ability to convey afferent (sensory) information about the state of the body’s organs to the central nervous system. Porges’ (1995) polyvagal theory specifies two functionally distinct branches of the vagus nerve that control the parasympathetic response in opposition to the sympathetic-adrenal system that mobilizes energy in times of stress. One branch, the ventral vagal complex (VVC), originates from the medullary nucleus ambiguous (NA) and provides efferents to supradiaphragmatic target organs (e.g., soft palate, pharynx, larynx, esophagus, bronchi, and heart). The NA also receives input from the trigeminal and facial nerves (Porges, 1995), suggesting influence of facial muscles in controlling cardiovascular, pulmonary, and parasympathetic output. The other branch originates from the medullary dorsal motor nucleus (DMX) and innervates subdiaphragmatic organs (e.g., stomach, intestines; Porges, 1995). Parasympathetic outflow is mediated largely by descending outputs from these nuclei and are under the direct influence of limbic regions associated with emotion-generation and conditioning of behavioral responses to emotional stimuli (Ulrich-Lai and Herman, 2009). One way that breath regulation may modulate parasympathetic output is through VVC efferents that regulate autonomic output, or indirectly through limbic inhibitory projections (Ulrich-Lai and Herman, 2009).

In yoga practice, breath regulation is a key tool for impacting physical and mental states, and vice versa (Faulds, 2005; Telles and Singh, 2013). In this context, diaphragmatic breath control is consciously mediated, but non-consciously influencing parasympathetic activation. The most common form of breath regulation during yoga practice is described as slow, deep, diaphragmatic, and paced along with each movement of the postures (Faulds, 2005); there are however, many other very specific practices that are described. A number of authors have proposed mechanisms for the self-regulatory properties of *pranayama* that support our model. In the late 1970s, *pranayama* was proposed to result in “a steadying of the mind” through the Hering–Berueter reflex (Pratap et al., 1978). Pauses after deep inhalation would result in excitation of baroreceptors (stretch receptors) in the lungs, providing feedback to vagal nuclei and facilitating increased vagal tone (Porges, 1995; described as a low-level brain mechanism in **Figure 1**). Action potentials would then travel along the vagus nerve to the lower pons, signaling inspiration. Increased stimulation of baroreceptors “may functionally alter some areas of the ascending reticular activating system, thereby suppressing sensory input to the cortex, bringing about a steadying of the mind” (Pratap et al., 1978). Other research groups supported this hypothesis and found that controlled breathing decreased chemoreflex response (i.e., a decrease in signaling of pH changes due to metabolic demand) and increased baroreflex sensitivity, both of which are vagus mediated (Spicuzza et al., 2000; Bernardi et al., 2001). An alternative explanation may be that change in respiratory rate results in changes in systolic blood pressure, which modulates the baroreflex (Bernardi et al., 2001). As described earlier, *ujjayi* breathing is a common technique used in combination with *asana* that is thought to strengthen the diaphragm, facilitating pulmonary gas exchange (O_2_/CO_2_) and circulation in the process (McCall, 2007). Postures that involve particular biomechanical changes in body position (e.g., “heart-opening poses”) could theoretically alter pulmonary ventilation, gas exchange, and cardiovascular function (Behrakis et al., 1983; Galanis et al., 2013; Karbing et al., 2013). It has further been suggested that mechanical forces from the body pushing against itself in particular postures may stimulate the hypothalamic–pituitary–adrenal (HPA) axis similar to massage (Rapaport et al., 2012). However, a recent study with yoga naive participants revealed that slow breathing with equal inspiration and expiration is as good in increasing blood oxygen saturation and baro reflex sensitivity as *ujjayi* on the exhalation (Mason et al., 2013). *Ujjayi* on both the inhalation and exhalation did not increase the baro reflex response at all when compared to normal breathing (Mason et al., 2013). This finding seems to be in conflict with a neurophysiological model of *ujjayi* breathing that hypothesized that stimulation of somatosensory vagal afferents and arterial baroreceptor result from the constricted breathing (Brown and Gerbarg, 2005b). Furthermore, holding the breath after inhalation or exhalation is proposed to enhance parasympathetic activity through the vagal afferents that project to the parabrachial nucleus, thalamic nuclei, cerebral cortex, and mesolimbic areas (Brown and Gerbarg, 2005b). Mason et al. (2013) speculate that their finding that *ujjayi* does not increase baro reflex sensitivity more than slow breathing, despite the prediction of the model of Brown and Gerbarg (2005b), might be due to increased effort in this pranayama. Breath regulation may also activate the hippocampus, hypothalamus, amygdala, and stria terminalis, which may subsequently improve autonomic function through coordinated conditioning of inhibitory feedback mechanisms associated with neuroendocrine release, emotional processing, and social bonding (Brown and Gerbarg, 2005b; Jerath et al., 2006; Telles and Singh, 2013). Other more challenging *pranayama* techniques such as the rapid exhaling in *kapalabhati* followed by breath retention or alternating nostril breathing are thought to similarly contribute to neurophysiological changes underlying self-regulation (as reviewed in Telles et al., 2011). A pilot study of HRV dynamics showed intermittent, very high amplitude oscillations in heart rate and high complexity in fluctuations of overall time series during slow breathing despite subjective experience of relaxation, over the course of meditative breathing and chanting exercises by advanced Kundalini yoga practitioners (Peng et al., 1999). These findings may tentatively support the notion that yoga practices including breathing train the ANS to be more dynamically adaptive to stressors (rather than statically quiescent), similar to the neurovisceral integration model of allostasis (Thayer and Sternberg, 2006).

### INTEGRATION OF TOP-DOWN AND BOTTOM-UP MECHANISMS

A result of yoga practice is improved integration among top-down and bottom-up mechanisms for self-regulation. Extinction learning is proposed to facilitate integration using bidirectional feedback between executive, viscerosomatic, and homeostatic processes. Our working model proposes the tools of yoga utilize associative mechanisms of extinction learning to re-condition the interactions across bodily systems during physical and/or emotional stress experienced on and off the yoga mat and re-orient cognitive, emotional, behavioral, and physiological output toward adaptive trajectories (indicated by dotted lines in **Figure 1**). Although there is likely to be influence by cognitive processes in an explicit way (see Ochsner and Gross, 2005), implicit sensory processes are conditioned to inhibit natural tendencies for physiological and emotional reactivity and more conscious resources are conserved for urgent, demanding, and exceptional matters (Quirk and Mueller, 2008). Interactions between vmPFC, vlPFC, and limbic areas (i.e., amygdala, hippocampus) have been shown to play both evaluative and non-conscious roles for extinction and behavioral inhibition and act as a hub for coordinating organism-wide visceromotor behavior (Ridderinkhof et al., 2004; Quirk and Mueller, 2008; Roy et al., 2012), thus playing supporting roles in facilitating new associations between body, mind, and behavior. Extinction learning in yoga practice involves integration between embodied movement, viscerosomatic, and homeostatic feedback, and parasympathetic control. We postulate that the extinction mechanism involves interactions among striatopallidal–thalamocortical circuitry, including cortical regions (i.e., dlPFC, ACC, orbitofrontal cortex), basal ganglia (striatum, pallidum), thalamus (mediodorsal nucleus), cerebellum, cortical sensory-motor nuclei (primary sensory, PMA, SMA), and fronto-temporal-parietal networks (Schacter, 1992; Quirk and Beer, 2006; Quirk and Mueller, 2008). These interactions are proposed to facilitate new, adaptive associations responsible for dictating adaptive and stable output.

Increases in tonic activity in striatopallidal–thalamocortical circuitry (including dopamine-containing nuclei) has been specified in implicit learning and automatized cognition (Liljeholm and O’Doherty, 2012) in parallel with top-down cognitive control of action and expectation. It is believed that feedback in iterative “loops” among cortico-subcortical circuits can act to “train” the cortex to produce more adaptive automatized and habitual motor output in the presence of a particular pattern of sensory information and conserve cognitive resources for ongoing task demands (Graybiel, 2008). As suggested earlier by van der Kolk (2006), our model also predicts that such circuitry is likely to become more active in experienced practitioners as the practice becomes more automatized and implicit. Morphometric and functional changes have been seen in this circuitry with fMRI studies of advanced meditators (Lazar et al., 2000; Holzel et al., 2008; Vago et al., 2013; Fox et al., 2014; Luders, 2014). Because of the proposed increases in integration across systems, implicit circuitry does not necessarily imply “unawareness,” but rather an ability to flexibly switch between more automatic processing of information and goal-directed manipulation of relevant information (Graybiel, 2008; Vago et al., 2013).

As a result, during the challenging aspects of yoga practice, appraisal processes become more efficient and implicit, resulting in stable attention and affect that may compete with old, previously conditioned stress reactivity, distraction, and affect dysregulation (Quirk and Mueller, 2008; Hartley and Phelps, 2010). This competition between old maladaptive habits and newer, more adaptive ones can be understood as an inhibition of the former by the latter, potentially facilitating adaptation of afferent–efferent and re-afferent processes (see Christoff et al., 2011). Through continued practice, inhibitory control mechanisms become integrated with autonomic, attentional, and affective systems, creating a functional and structural network for self-regulation (Thayer and Lane, 2000). The strengthening of habitual inhibitory control via increased vagal tone is proposed to activate feedback and feed-forward circuits that re-condition autonomic reactivity across contexts (e.g., off the yoga mat), similarly described in the neurovisceral integration model of allostasis (Thayer and Lane, 2000; Thayer and Sternberg, 2006). Such neurovisceral integration is believed to dampen the release of neurochemical mediators (i.e., “stress hormones”) across brain and bodily systems in an adaptive controlled process that extinguishes the prolonged sympathetic arousal and associated cognitive and emotional dysregulation that is habitually conditioned as a response (Thayer and Sternberg, 2006).

Another integrative tool for yoga’s role in self-regulation is proposed to function through its increased attention to afferent information and emphasis toward processing bottom-up information. This shift is thought to alter integration between the bottom-up and top-down processes and can be described from an interoceptive predictive coding framework (Seth et al., 2011; Seth, 2013). Briefly, according to this framework, goal-directed or inferred states result in the top-down generation of predictive models of interoceptive signals. These models are then compared to the actual afferent bottom-up input, resulting in a prediction error that describes the difference between the top-down prediction and the bottom-up input. This prediction error can then be resolved either through active inference which includes changing the environment through motor and autonomic control, or through perceptual inference, which entails updating the model to accommodate the afferent input (Seth, 2013). If this prediction error would get resolved by active inference, matching afferent input would be generated and the conditioning would be maintained, while perceptual inference would result in extinction. Greater precision of afferent signals as the result of increased sensory attention, leads to perceptual inference. Yoga is proposed here to facilitate perceptive inference and thereby lead to extinction of maladaptive behaviors as described above.

A natural outcome of yoga practice has been demonstrated to be increased cardiovascular tone, musculoskeletal strengthening, balance and flexibility (see Yoga-facilitated output of **Figure 1**), benefits that may relate directly to the exercise component of practice (Ross and Thomas, 2010). In fact, many studies of yoga employ exercise as a comparison group (Ross and Thomas, 2010; Streeter et al., 2010), inferring that yoga may improve mental health at least in part via similar mechanisms as does exercise. The aerobic component of yoga practice facilitates beneficial changes in hemodynamic, hormonal, metabolic, neurological, and respiratory function (Ross and Thomas, 2010). These positive changes are the result of bottom-up mechanisms that increase metabolic efficiency of oxygen by the brain and body and maximize cardiac output (the volume of blood ejected by the heart per minute, which determines the amount of blood delivered to the exercising muscles; Fletcher et al., 1996). Ross and Thomas (2010) report in their review of 12 existing studies that yoga has been shown to be as effective or superior to exercise on nearly every outcome measured in healthy individuals (Ross and Thomas, 2010). There is some evidence that yoga postures may specifically improve symptoms of depression, stress, and anxiety (see Field, 2011; Li and Goldsmith, 2012). The influence of related physical exercise on psychological well-being has been fairly well-established (Hassmen et al., 2000; Penedo and Dahn, 2005), while the relationship between physical fitness and self-regulation is less clear. Although it remains unclear how yoga specifically differs from exercise, we hypothesize that postures in parallel with cardiovascular challenge may lay an essential foundation of implicit regulation on the yoga mat, facilitating increased vagal tone and equanimity during increased cardiovascular tone off the yoga mat.

In summary, based on existing research on stress modulation and conceptual understandings by which yoga is intended to function, we propose a model (**Figure 1**) that may influence self-regulatory systems in the context of physical and emotional stress that involves high-level and low-level brain networks and the associated top-down and bottom-up processes. This systems network model includes the major limbs of yoga, represented as a skillset of four process tools: ethics, meditation, breath regulation, and sustained postures. As depicted in the model, cognitive, emotional, behavioral, and autonomic output in response to a stressor is modulated by a number of regulatory processes (yellow boxes) proposed to be influenced by the process tools (limbs of yoga, blue boxes). A stress response is often accompanied by cognitive, emotional, and behavioral output that includes emotional reactivity, negative appraisal, and rumination (Chrousos and Gold, 1992a). In addition, autonomic output such as vasoconstriction, pain and/or tension, and inflammation often accompany maladaptive stress responses (Chrousos and Gold, 1992a; see solid black arrows). In chronic forms of such stress responses, negative, long-term consequences on health across bodily systems are often the result. Our model proposes that yoga facilitates adaptive output (dotted lines), including long-term psychological and physical well-being, musculoskeletal strengthening, and prosocial behavior, through four primary factors in the context of stress: (1) an emphasis on interoception and bottom-up input, (2) more efficient bidirectional feedback and integration with top-down processes, (3) increased phasic inhibition (red lines) of maladaptive forms of emotional, cognitive, and behavioral output (e.g., reactivity, negative appraisal, rumination) as well as autonomic output (e.g., vaso- and pulmonary constriction, inflammation, and muscle tension/pain), and (4) perceptual inference rather than active inference for improved prediction and error correction processes. These four factors optimize self-regulation and improve the communication and flexibility by which top-down and bottom-up processes inform behavioral output in the context of physical and emotional stress. Through repeated yoga practice, there is a resulting skillful optimization of autonomic control in response to stressors on and off the yoga mat – keeping arousal at lower levels during stress-mediated challenge, maintaining positive appraisal and reinforcement, helping the practitioner stay relaxed with less effort, and facilitating rapid recovery of bodily systems under stress. A number of cognitive, emotional, behavioral, and autonomic mechanisms are proposed along with the underlying high- and low-level brain networks that support such mechanisms.

A central executive network supports top-down mechanisms of attentional control and working memory allowing stable engagement with appropriate exteroceptive/interoceptive input for proper goal-directed behavior followed by self-correction if needed. A FPCN supports executive monitoring, meta-awareness, reappraisal, and response inhibition mechanisms. A moral cognition network supports positive forms of re-appraisal, as well as motivation and intention setting associated with self-care and prosocial behavior. The dorsal attention network helps to support attentional orienting, and engagement. HPA axis communication with brainstem vagal efferents support parasympathetic control, diaphragm strengthening, and homeostasis across systems. A striatopallidal–thalamocortical network is responsible for facilitating extinction learning and reconsolidation of maladaptive habits into behavior that is aligned with intentions and outcomes into adaptive habits.

## EMPIRICAL EVIDENCE FOR THE PROPOSED MODEL AND THAT YOGA PROMOTES SELF-REGULATION ACROSS COGNITIVE, EMOTIONAL, BEHAVIORAL, AND AUTONOMIC DOMAINS

The theoretical model described above suggests that yoga practice may have salutary effects on self-regulation of cognitions, emotions, and behaviors through myriad pathways. Although this model is quite thorough and integrates knowledge across historical and contemporary theories of yoga and the current scientific literature associated with self-regulation, the empirical study of their linkages is sparse at this time. Below, we report the extant observational and intervention studies that have examined associations between yoga practice and self-regulation in a variety of populations.

### EVIDENCE THAT YOGA AIDS IN COGNITIVE REGULATION

There is growing scientific evidence that yoga practice has an effect on cognition and processes underlying its regulation. For example, several studies have examined whether yoga can improve attention in children and adults. Ten days of uni-nostril or alternate nostril breathing resulted in increased spatial memory in children (10–17 years; Naveen et al., 1997). Adults showed improved performance on the letter-cancelation task after right and alternate nostril breathing (Telles et al., 2007). Kapalabhati and breath awareness have recently been shown to reduce optical illusion (Telles et al., 2011). An early randomized controlled trial of 14 children diagnosed with attention deficit hyperactivity disorder (ADHD) and on medication compared 20 sessions of yoga to an active control group, finding that those in the yoga group improved more in parent-rated ADHD scores (Jensen and Kenny, 2004). More recent studies of children with ADHD or attention problems have also shown positive effects (e.g., Harrison, 2004; Peck et al., 2005; Haffner et al., 2006). Studies have shown that memory and concentration increase in groups other than children with ADHD as well. One study found improvement in memory in Brazilian military recruits who participated in yoga as well as exercise compared to recruits in an exercise-only condition; effects were particularly strong for those under stressful conditions, but the improved memory persisted at a 6 month follow-up (Rocha et al., 2012). A study of adolescents found that a 7-week yoga program improved memory and concentration (Kauts and Sharma, 2009), and a study demonstrated acute improvement on speed and accuracy in math computations in a sample of 38 adults who participated in a 20-min Tai chi/yoga class; the authors attributed the improvement to the observed increased relaxation in the sample, although they did not test for mediation (Field et al., 2010). On a more physiological level of bottom-up sensory processing, an EEG study revealed decreased P300 latency following alternate nostril breathing and increased P300 peak after breath awareness in an auditory discrimination task. These findings were interpreted as decreased time needed for discrimination and increased availability of neural resources respectively (Joshi and Telles, 2009). A recent fMRI study with older adults found that age related decline in fluid intelligence was off-set in long-term yoga practitioners and that yoga practitioners had more efficient functional brain networks than carefully matched controls (Gard et al., 2014).

Many of these preliminary studies demonstrate improved cognition and suggest increased integration between explicit and implicit processes that regulate such cognitive and perceptual abilities. It should be noted that not all studies have found positive effects of yoga on cognitive regulation in either acute (e.g., Telles et al., 2012) or long-term yoga interventions (e.g., Chaya et al., 2012). In general, the literature testing the effects of yoga on cognitive regulation effects is preliminary, with small samples and lack of appropriate control groups. However, these initial findings are consistent with the notion that yoga can aid in better cognitive regulation.

### EVIDENCE THAT YOGA AIDS IN EMOTION REGULATION

Literature demonstrating yoga’s effects on emotion-regulation strategies is limited, but accumulating, both for acute (i.e., immediately following a yoga practice) and longer-term outcomes. One study examining the acute effects of a single yoga session on healthy women found that relative to a control group (who concentrated on reading a newspaper), women who experienced a yoga session reported less emotional lability, excitability, and aggressiveness. Further, they reported a lower tendency to cope with stress through aggression and self-pity and a higher tendency to cope through downplaying (reappraising) a situation (Schell et al., 1993). A yoga intervention with college students demonstrated that yoga increased students’ self-compassion and emotion regulation skills (reductions in the difficulty with emotion regulation scale) and increased non-judgmental self-reflection (Sauer-Zavala et al., 2012). Similarly, in an adolescent sample, preliminary trends indicated that yoga during physical education may improve emotion regulation strategies relative to physical education-as-usual, perhaps through increasing emotion awareness (Noggle et al., 2013).

Yoga practice may also improve cognitive reappraisal (Garland et al., 2011), a form of emotion regulation that involves an ability to change the trajectory of an emotional response by reinterpreting the meaning of the stimuli. In a recent study, practitioners of Sudarshan Kriya and related practices (SK&P) were presented aversive pictures and asked to cognitively change their appraisal of the affective meaning of them by coming up with an alternative more positive interpretation of each picture. Relative to a non-yoga-practitioner control group, long-practicing yogis demonstrated a longer-lasting change in reduced magnitude of P300 event-related and late-positive potentials, indicating greater emotion regulation (Gootjes et al., 2011). Furthermore, Sudarshan Kriya has been reported to be beneficial for the treatment of depression, anxiety, stress, and post-traumatic stress disorder (for a review see Brown and Gerbarg, 2005a).

In addition to yogic breath regulation, yoga postures may also influence emotion regulation at a more physiological level. Breathing patterns of rapid inhalation and slow exhalation at an overall reduced respiration rate has been shown to decrease heart rate, skin conductance, and psychological arousal in a threatening situation (Cappo and Holmes, 1984). Preliminary data investigating different asanas on peripheral physiology suggests that basic body posture (spinal flexion, extension, or neutrality) may influence psychophysiological reactivity to interoceptive threat, possibly depending upon affective context (Wielgosz et al., 2012).

### EVIDENCE THAT YOGA AIDS IN BEHAVIORAL REGULATION

A small amount of research suggests that yoga can help in behavioral regulation. One recent study found that a 10-week, twice-weekly yoga intervention with previously inactive participants increased their longer-term adherence to a physical activity regimen, indicating that yoga can boost one’s ability to regulate a fairly difficult behavior, adherence to physical activity (Bryan et al., 2012). Another recent study of first- and second-year medical students enrolled in an elective yoga and mindfulness course showed that goal-directed regulation of behavior improved pre- to post-, according to self-report (Bond et al., 2013).

Investigations of nicotine addiction have shown that yoga may positively influence behavioral regulation. A study of women in cognitive–behavioral treatment for smoking cessation compared a yoga therapy condition to a general health and wellness program control condition. Women receiving yoga had higher 7-day smoking abstinence rates than controls at the end of the intervention, and abstinence remained higher among yoga participants through the 6 month follow-up (Bock et al., 2012). This increased behavioral control may be at least partly due to decreased cravings. Another study found that daily smokers assigned to either a brief yoga intervention or an exercise intervention, relative to a passive control, reported a decrease in craving to smoke. Further, while the exercise group reported lower craving in response to smoking cues, those who had received yoga reported a general decrease in cravings (Elibero et al., 2011). Although few in number, these studies are consistent with the notion that yoga may be useful in facilitating long-term regulation of behaviors that require considerable self-regulation, such as physical activity or smoking abstinence.

Yoga dose (amount, frequency, length of practice) may also be important for behavioral regulation. In a convenience sample of yoga practitioners, the amount of yoga practiced was modestly and inversely related to the use of dysfunctional coping, a coping style characterized by disengagement, venting, and substance use, suggesting that yoga may help to mitigate dysfunctional coping behavior (Dale et al., 2011). In a small sample, a yoga workshop increased participants’ ability to recognize and respond to emotional states and to reduce mood instability, impulsivity, recklessness, and self-destructive behaviors (Dale et al., 2009).

## CONCLUSION

There is emerging evidence from the extant literature to support the beliefs that modern adaptations of yoga practice are beneficial for mental and physical health. Here, we delineate very specific components of yoga practice (ethics, postures, breath regulation, and meditation) that are rooted in a historical framework and employed in varying degrees in contemporary contexts as a model for understanding how yoga may achieve its benefits – facilitating self-regulation and resulting in psychological and physical well-being. Beginning with a brief scholarly perspective of yoga history and philosophy, we present the core psychological, cognitive, and neuroscientific understandings of yoga in the context of a spectrum of physical and mental stress. Our proposed framework and systems network model integrates a great deal of theory and research regarding both bottom-up and top-down self-regulatory processes and the ways through which yoga may contribute to self-regulation across cognitive, emotional, behavioral, and physiological domains. Yet, we acknowledge that we were not able to capture all the likely relevant dimensions.

In summary, we propose that yoga practice facilitates self-regulation via an ethically motivated monitoring and control process that involves initiation and maintenance of behavioral change as well as inhibiting undesired output by both higher-level and lower-level brain networks in the face of stress-related physical or emotional challenge. We propose yoga practices emphasize a shift toward bottom-up interoceptive processing and the integration of self-regulatory bottom-up and top-down processes across bodily systems (including cardiovascular, neuroendocrine, and musculoskeletal). Particular mechanisms from high-level brain networks are proposed, including intention/motivational goal setting, attentional control, meta-awareness, response inhibition, working memory, and cognitive reappraisal; mechanisms from low-level brain networks are also proposed, including parasympathetic control, improved baroreceptor functioning, increased vagal tone, strengthening of the diaphragm, extinction learning, and early forms of attentional orienting and engagement. The mechanisms described provide a working model that integrates autonomic, cognitive, behavioral, and affective processes into a multi-systems framework for adaptive functioning, all of which serves to promote acute and long-term effects on well-being and mental and physical health. Through practice on the yoga mat, the proposed mechanisms are likely to be more successfully generalized into individuals’ lives off the mat, equipping the practitioner with skills to enable adaptive autonomic nervous system functioning, and cognitive–emotional–behavioral processes that are more flexible and adaptive to emotional and homeostatic perturbations throughout daily life.

## FUTURE DIRECTIONS

This framework serves as a starting point for understanding the growing research findings linking yoga with psychological and physical well-being, prosocial and ethical behavior, and musculoskeletal strengthening in terms of self-regulation. While it is promising that the framework accommodates current research findings, systematic testing of it is still required. As a result, and in line with our aims, the framework proposed is suitable to guide future research. Where possible, we have presented empirical literature examining the hypothesized links. However, for many of these links, very little research is available and much theory remains speculative. Although a sizable literature links yoga practice with psychological health, few studies have assessed mediators of the effects of the individual components of yoga on psychological health or examined how yoga practice in general affects self-regulatory processes.

Future research may elaborate on specific process tools and process outcomes that we propose improve the efficiency and adaptive nature of habitual forms of cognition, emotion, and behavior across systems of mind–brain–body functioning at both top-down and bottom-up levels. For simplicity, in **Figure 1** we did not map relative contributions of process tools onto process outcomes. For example, parasympathetic activation may be heavily influenced by breath regulation, but also to lesser degrees by sustained postures and meditation; and these influences may change depending on the particular techniques used and an individual’s psychological and physical state. Furthermore, the influence of yogic tools on several of the process outcomes proposed in this model has not been directly studied.

Future studies should go beyond cross-sectional designs (e.g., novice vs. long-term practitioners) and focus on longitudinal designs with appropriate active control designs in order to rule out the multitude of potential third variables underlying someone’s status as a long-time practitioner, and whether those aspects that differentiate long-term practitioners mediate any noted improvements in psychological well-being. Development of a testable online yoga research database analogous to BRAINnet’s use of Brain Resource International Database (www.brainnet.net/about/brain-resource-international-database/) may afford such long-term needs especially with a focus on tracking people over time.

Yoga research is made more complex because of the many different process tools (“limbs”), each of which may have differential effects on various forms of self-regulation. Effects of some process tools have been minimally studied. For example, we were able to find only one study that examined the effects of explicit inclusion of *yamas* and *niyamas* in a yoga intervention (Smith et al., 2010). The need to assess the different contributions of particular process tools of yoga and to examine their unique contributions to outcomes is recognized among yoga researchers (e.g., Sherman, 2012). Toward this end, one group of researchers has been developing a new instrument with which yoga researchers can describe their interventions on key dimensions such as challenging asana, restorative asana, meditation, breathwork, spirituality, ethical principles, etc. (Park et al., 2014). Once completed, this instrument may provide the necessary quantification of yoga’s various components to allow researchers to examine the unique effects of each on self-regulation. However, it may be that some of yoga’s most potent effects on self-regulation are due to a synergy of multiple process tools of yoga, rendering dismantling studies of yoga practice potentially less useful for understanding its impacts on self-regulation. Yoga practice may be particularly helpful in promoting self-regulation for certain populations or conditions (e.g., major depression, ADHD, chronic pain; see Balasubramaniam et al., 2012) with known abnormalities in CNS and/or behavior that may allow for more targeted testing of the components of this model; this notion reflects the yogic perspective of the practice of meeting people where they are. In addition, different components of yoga practice may be better suited for different people, depending on individual differences. Such issues remain to be explored within the context of self-regulation and may be important components to include in recommended complexity-based analyses.

Such synergetic effects may also be non-linear requiring more sophisticated analyses that capture such complexity. For example, studies that employ non-linear dynamical models of self-organization and emergence may capture not only the integration of top-down and bottom-up neurological processes involved in the proposed self-regulatory mechanisms of yoga, but also the dynamic relationships of these processes to psychosocial and clinical outcomes (Pincus, 2012). Study designs that incorporate measures of heterogeneity, such as parsimony phylogenetics to define randomization groups and pattern or network analysis instead of analysis of averages, may respectively result in stronger comparison groups and more accurate representation of results than traditional randomized controlled trial designs (Abu-Asab et al., 2012). For example, in a study of tai chi in older adults with peripheral neuropathy, a complexity measure of postural control based on multiscale entropy analysis was able to detect significant changes that were correlated with improvements in function as opposed to linear analyses of traditional outcomes which failed to detect these changes (Manor et al., 2013).

An additional, important dimension that requires further elaboration includes social outcomes of yoga practice, such as the prosocial behavior briefly referenced in this paper, as well as social influences on self-regulation. Team sports and exercise literature suggests that group identity can influence motivation and consistency of practice (Fraser and Spink, 2002; Emmons et al., 2007). Given that much of modern yoga practice occurs in a group setting, it follows that similar influences of group identity on behavioral self-regulation may occur. Yet distinct from exercise, yoga may foster internalized experiences of physical activity within a group setting, whereas exercise (and most other social interactions) may orient more externally using extensive verbal and visual body language cues. Not only is a thoughtfully developed social process of this model needed, but it may also help inform the best types of control groups in future studies of yoga. Especially when studying social and behavioral aspects of our model of self-regulation, a mixed methods design approach may be helpful. Systematically capturing both qualitative data on participant experiences and quantitative data on mechanisms and outcomes, and then rigorously integrating the analysis and interpretation of these different data streams, may, for example, allow novel insights into the heterogeneity of response from a complexity analysis or a deeper understanding of those who may be more responsive to a particular yoga treatment than others.

We are hopeful that our model will prove useful to researchers examining the multiple systems through which yoga can affect self-regulation. It is clear that yoga practice has both top-down and bottom-up effects and a better understanding of these pathways and their integration will provide important advances in our understanding of human functioning as well as promote more effective interventions for improving human health and well-being.

## Conflict of Interest Statement

The authors declare that the research was conducted in the absence of any commercial or financial relationships that could be construed as a potential conflict of interest.

## Footnotes

1 In the following summary of yogic concepts that may relate to self-regulation, we included key yogic terms italicized in Sanskrit for historical reference.
